# Supplementary material for: EGFR testing and erlotinib use in non-small cell lung cancer patients in Kentucky
Source: PLoS One. 2020 Aug 18;15(8):e0237790. doi: 10.1371/journal.pone.0237790 (PMC7433873; doi:10.1371/journal.pone.0237790)
Supplement: Attachment — Submitted filename: Response to reviewers.doc.docx [file pone.0237790.s001.docx]

Author response to reviewer comments

Reviewer #1: Since both reviewers raised the concern of novelty and relevance of the study, please ask the authors to explain why they did not choose to expand the analysis using the Kentucky Cancer Registry to years beyond 2011 to reflect the changing practice patterns in EGFR-driven NSCLC, rather than simply rephrasing their discussion. If this is feasible, it should done.

**Author response:** The linked claims database was created with a funded research collaboration that covered the time period reported, therefore it is not feasible to update the analysis.

The following was added to the limitations paragraph: “Linked claims were only available for the time period reported, so while these results do not reflect current practice patterns, the study presented the opportunity to study the implementation of a single precision medicine intervention without competing interventions. We hypothesize that precision medicine interventions continue to lag in rural communities and this highlights the need for further study.”

Reviewer #2: Table 4: Since EGFR screening is associated with younger non-smoking women (most likely a surrogate for selecting appropriate patients for testing), is it possible that younger non-smoking women were more likely to have private insurance and lower poverty? Have you performed any statistical tests to see if these are associated? If so, it is possible that being a young non-smoker woman is the most important variable associated with testing and not necessary access to testing.

**Author response:** Table 4 shows the primary effect of significant factors associated with receiving erlotinib in a logistic regression analysis. The model identified independent association of each factor for the outcome variable. Hence the effects of insurance and poverty are significant regardless the status of age, gender or smoking. No changes were made to the manuscript.

Discussion paragraph 5: The conclusion that that the improved survival with EGFR testing and erlotinib "is likely due to those patients receiving better overall healthcare, relating to care access, insurance coverage or poverty status" is flawed and not supported by your data. Overall survival is most likely better because the patients that received EGFR testing were more likely to have an EGFR mutation and thus obtained benefit from erlotinib. You state that this is not the case because only 10-15% would be positive for the mutation. This 10-15% is for the general public. It is most likely that those receiving EGFR testing in your population have a high chance of having an EGFR mutation given they they are predominately younger non-smoking women. Also, a population enriched with 10-15% of patients with EGFR mutations receiving erlotinib can still be responsible for the overall survival benefit. If you want to make a conclusion that access to care leads to better survival that is fine. But use the metropolitan vs rural and medicaid/medicare vs private insurance analysis instead of the EGFR/erlotinib analysis to support this conclusion.

**Author response:** The 5^th^ paragraph of the discussion was revised as follows:

As expected, younger age, female gender, lower stage, and less comorbidities were associated with improved survival. Other factors associated with better survival included having private insurance and living in a non-Appalachia, metropolitan area. Since testing itself should not impact survival, this is likely due to those patients receiving better overall healthcare, related to better care access to care or better insurance coverage.
